# Integrative lncRNA–mRNA co‐expression network analysis identifies novel lncRNA E2F3‐IT1 for rheumatoid arthritis

**DOI:** 10.1002/ctm2.325

**Published:** 2021-02-24

**Authors:** Long‐Fei Wu, Xing‐Bo Mo, Jia‐Hui He, Pei He, Xin Lu, Hong‐Wen Deng, Fei‐Yan Deng, Shu‐Feng Lei

**Affiliations:** ^1^ Center for Genetic Epidemiology and Genomics School of Public Health Medical College of Soochow University Suzhou P. R. China; ^2^ Jiangsu Key Laboratory of Preventive and Translational Medicine for Geriatric Diseases Soochow University Suzhou P. R. China; ^3^ Center of Bioinformatics and Genomics Department of Global Biostatistics and Data Science Tulane University New Orleans Louisiana USA

Dear Editor,

Our previous study has reported that DNA methylation serves as an important epigenetic factor of gene–environment interaction, which contributes to pathogenesis of rheumatoid arthritis (RA).^1^ To investigate the role of another epigenetic factor (long noncoding RNA, lncRNA) in RA pathogenesis, we integrated lncRNA and mRNA transcriptomic information, constructed lncRNA→mRNA→RA regulatory network by performing co‐expression networks analysis and causal inference test, and explored functional roles of the highlighted lncRNA E2F3‐IT1 (E2F3 intronic transcript 1) in RA (Figure S1).

This study first isolated peripheral blood mononuclear cells (PBMCs) from RA patients (N = 25)^2^ and age‐ and sex‐matched controls (N = 18) (Table 1A), and tested 22,774 lncRNAs and 25,004 mRNAs expressions through human microarray (Figure 1A). Differential expression analyses identified a total of 402 lncRNAs and 832 mRNAs (fold‐change > 2 and false discovery rate < 0.05) as potential targets for subsequent analyses (Table S1). Since the functions of lncRNAs are largely unknown, we performed the weighted gene co‐expression network analysis (WGCNA)^3^ by simultaneously incorporating information of the above differential expressed genes, and two interesting co‐expression modules were constructed, named yellow and magenta (Figure 1B). The yellow module has the highest module significance, indicating that the yellow module genes were more likely associated with RA. GO functional enrichment analysis showed that the genes in the yellow module were enriched in “regulation of innate immune response” and “regulation of gene expression” (Table S2). From the yellow module, a total of 31 hub lncRNAs and 30 hub mRNAs (correlation coefficient *r* ^2^ ≥ 0.8) were selected (Table S2) to construct the regulatory chain of lncRNA (causal factor) →mRNA (mediator) →RA (outcome) through causal inference test (CIT) analysis.^4^ A total of 191 significant causative regulatory chains were identified including 20 lncRNAs and 21 mRNAs (Figure 1C and Table S3).

**TABLE 1:** Basic characteristics of the study subjects and the expression levels of the selected mRNA and lncRNA in PBMCs in the validation sample

**(A) Basic characteristics of the study subjects**				
	**Discovery group**		**Validation group**	
**Variable**	**RA patient (*n* = 25)**	**Healthy control (*n* = 18)**	**RA patient (*n* = 35)**	**Healthy control (*n* = 35)**
Gender	Female	Female	Female	Female
Age (year)	45.6 ± 9.84	47.11 ± 14.09	46.44 ± 10.99	47.57 ± 13.99
BMI (kg/m^2^)	22.07 ± 3.31	22.32 ± 2.79	22.24 ± 3.67	22.32 ± 2.79
DAS28	4.46 ± 0.99	n.d.	5.08 ± 1.32	n.d.
CRP (mg/L)	13.51 + 16.74	n.d.	18.37 ± 31.17	n.d.
ESR (mm/h)	42.61 ± 27.5	n.d.	48.29 ± 29.46	n.d.
TJC	8.64 ± 6.49	n.d.	10 ± 7.7	n.d.
SJC	5.48 ± 3.83	n.d.	6.79 ± 5.43	n.d.


**(B) The expression levels of the selected mRNA and lncRNA in PBMCs in the validation sample**				
**lncRNA**	**Chromosome**	**Fold change**	***p*‐Value**	**Class**
CYTOR	17q11.2	1.08	0.67	Intergenic
DQ593252	11q12.3	1.21	0.05	Intergenic
E2F3‐IT1	6p22.3	1.73	0.03	Intronic
uc.265	9q31.1	1.35	0.02	Antisense
INE2	Xp22.2	1.43	0.049	Antisense
**mRNA**				
DDX58	9p21.1	1.06	0.67	DExD/H‐box helicase 58
IFI16	1q23.1	1.09	0.35	Interferon gamma inducible protein 16
LDLR	19p13.2	2.13	<0.001	Low‐density lipoprotein receptor
PLSCR1	3q24	‐1.67	<0.001	Phospholipid scramblase 1
PARP9	3q21.1	1.39	0.015	Poly(ADP‐ribose) polymerase family member 9

Abbreviations: BMI, body mass index; DAS28,28 joint Disease Activity Score; CRP, C reactive protein; ESR, equivalent series resistance; TJC, tender joint count; SJC, swollen joint count; n.d., not determined.

Notes: Variables were expressed as the mean ± SD. *p‐*Value represents the significance for the difference between RA patients(*n* = 35) and healthy controls(*n *= 35).

**FIGURE 1:**
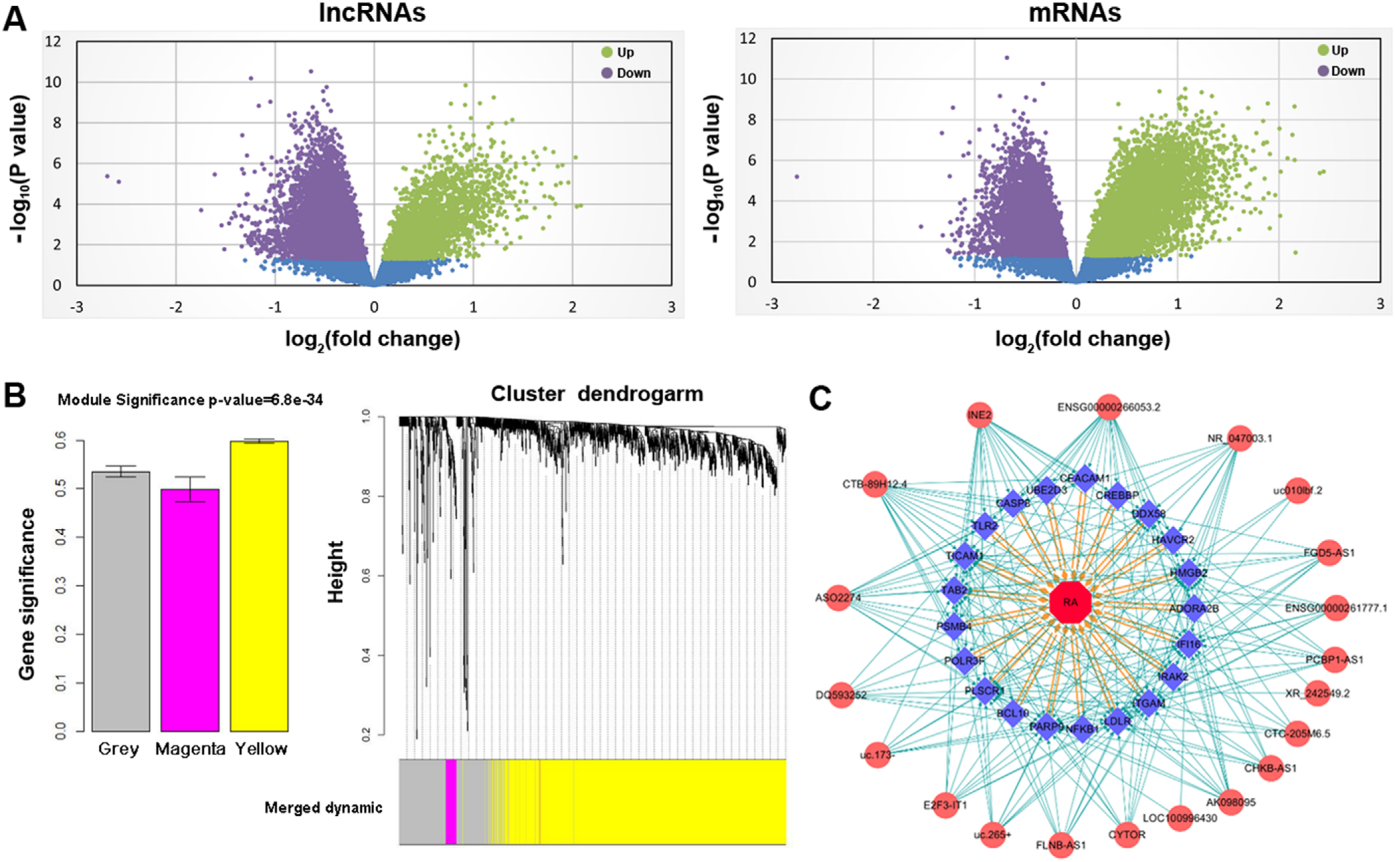
Integrative lncRNA and mRNA co‐expression network analysis and causal inference test (CIT) analysis. (A) Volcano plot of lncRNA and mRNA expressed in RA patients and healthy controls. (B) Gene modules of differentially expressed mRNAs and lncRNAs in PBMCs identified by WGCNA. Left: Diagram of correlation of module's color and RA. The colored column indicates modules and the *Y*‐axis represents gene significance. Right: Clustering dendrograms of PBMCs. System clustering tree was built based on PBMCs dataset. Three kinds of colors present three modules. (C) Significant lncRNA→mRNA→RA regulatory chains identified by CIT

To verify hub lncRNAs and mRNAs in the causative regulatory chains, five lncRNAs (*CYTOR*, *UC.265*, *DQ593252*, *E2F3‐IT1*, and *INE2*) and five mRNAs (*DDX58*, *IFI16*, *LDLR*, *PLSCRI*, and *PARP9*) were selected for validation in another sample including 35 RA patients and 35 healthy controls. The RT‐qPCR results showed that three lncRNAs (*UC.265*, *E2F3‐IT1*, and *INE2*) and three mRNAs (*LDLR*, *PLSCRI*, and *PARP9*) in PBMCs were also differentially expressed in the validation sample (Table 1B). The constructed lncRNA→mRNA→RA causative regulatory chains for the three validated lncRNAs and three validated mRNAs showed that *LDLR* serves as a significant mediator between lncRNAs (e.g., *UC.265*, *E2F3‐IT1*, and *INE2*) and RA disease outcome (Table S4). Among the validated differentially expressed lncRNAs, E2F3‐IT1 presented higher fold‐change and smaller statistical *p*‐values and hence was further assessed for its functional roles in the pathogenesis of RA.

RA is characterized by the breakdown of immunological tolerance. Aberrant T‐cell activation has been recognized as the central event in chronic inflammation and synovial hyperplasia.^5^ Next, lncRNA knock‐down cells (E2F3‐IT1‐SH) were constructed by stably transfecting lentiviral vectors harboring E2F3‐IT1 target sequence into Jurkat T cells. Comparing with negative control (E2F3‐IT1‐NC) cells, lncRNA E2F3‐IT1‐SH cells presented significantly decreased lncRNA E2F3‐IT1 expression (Figure 2A and B), suggesting that E2F3‐IT1 was successfully targeted and silenced in Jurkat T cells. To determine whether lncRNA E2F3‐IT1 affects cell proliferation, cell number was examined by using the CCK8 assay. Compared with negative control cells, knockdown E2F3‐IT1 significantly inhibits cell proliferation (Figure 2C). In addition, an increased percentage of apoptotic cells was observed in lncRNA E2F3‐IT1 knockdown cells through flow cytometry analysis (Figure 2D). Furthermore, we found that knockdown E2F3‐IT1 could lead to an increased percentage of S‐phase cells and a reduced percentage of G2‐phase cells through the cell cycle assay (Figure 2E). LncRNA E2F3‐IT1 co‐expression genes including *LDLR*, *PLSCR1*, and *PARP9* were also successfully validated in vitro by using E2F3‐IT1‐SH cells (Figure 2F). We then examined the effect of lncRNA E2F3‐IT1 on T‐cell activation stimulated with immune activator phorbol‐12‐myristate‐13‐acetate (PMA). Under PMA stimulation, the inflammatory cytokines, such as IL‐1, IFN‐γ, and TNF‐α, presented downregulated expression in E2F3‐IT1‐SH cells as compared with E2F3‐IT1‐NC cells (Figure 2G). Flow cytometry analysis showed that the expression of antigen CD69 (an early T cells activation biomarker) was increased by PMA induction in E2F3‐IT1‐NC cells, which was attenuated by knockdown of lncRNA E2F3‐IT1 (Figure 2H). These results taken together indicated that lncRNA E2F3‐IT1 may be involved in RA pathogenesis by affecting T‐cell growth and activation.

**FIGURE 2:**
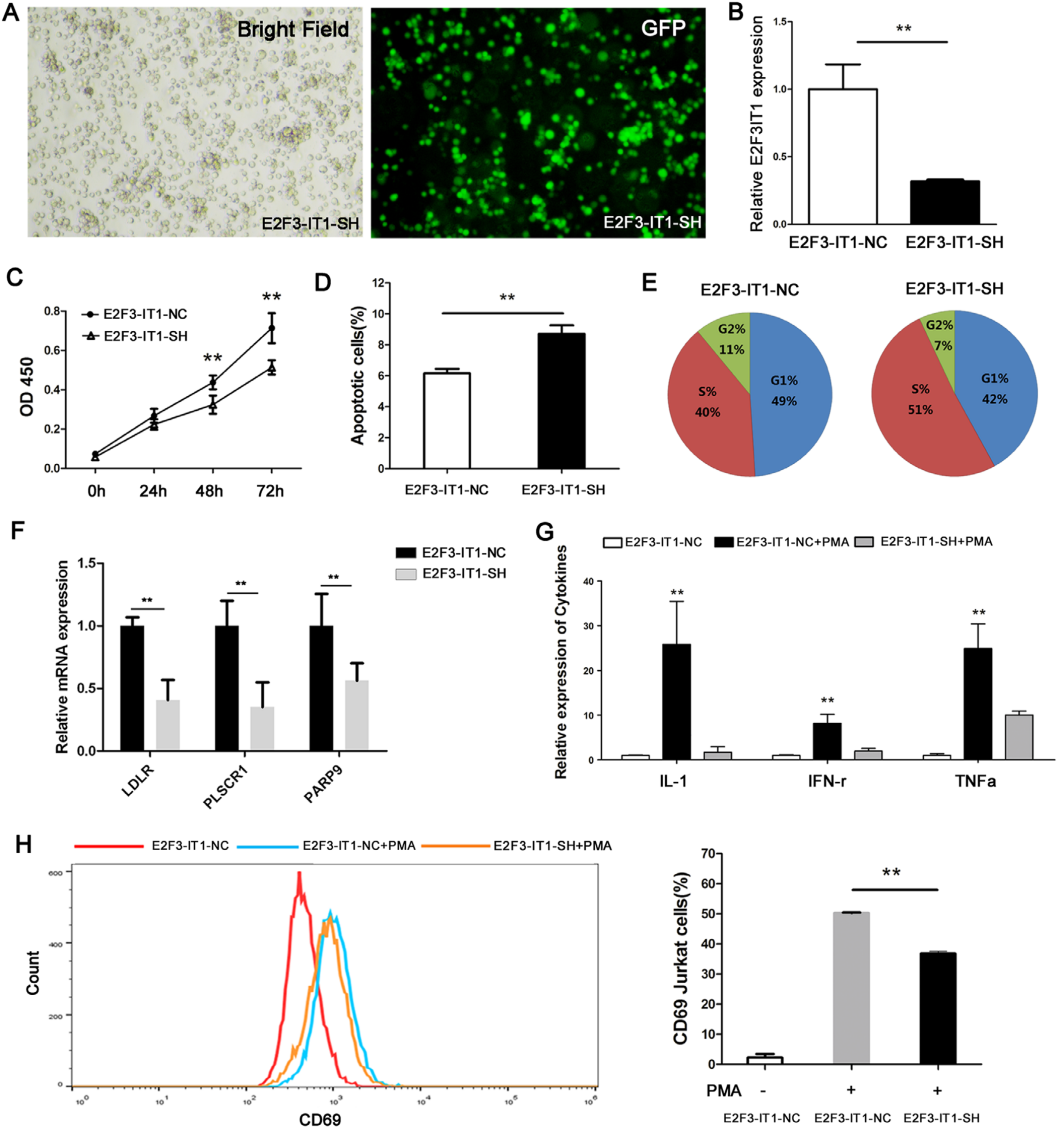
The functional role of lncRNA E2F3‐IT1 in Jurkat cells in vitro. (A) E2F3‐IT1‐SH Jurkat cells in bright field (left) and fluorescent field (right). (B) Relative expression of lncRNA E2F3‐IT1 in E2F3‐IT1‐SH and E2F3‐IT1‐NC cells detected by RT‐qPCR. (C) OD450 values of E2F3‐IT1‐SH and E2F3‐IT1‐NC Jurkat cells in cell proliferation assays. (D) Percentages of apoptotic Jurkat cells detected by annexin V/7AAD double staining through flow cytometry. (E) Percentages of stage‐specific cells within the cell cycle for E2F3‐IT1‐SH and E2F3‐IT1‐NC. (F) Relative expression of *LDLR*, *PLSCR1*, and *PARP9* genes in E2F3‐IT1‐SH and E2F3‐IT1‐NC cells examined by RT‐qPCR. (G) Relative expression of IL‐1β, IFN‐γ, TNF‐α cytokine in PMA‐stimulated E2F3‐IT1‐SH and E2F3‐IT1‐NC cells. (H) Expression of cell activation marker CD69 in PMA‐stimulated E2F3‐IT1‐SH and E2F3‐IT1‐NC Jurkat T cells were assessed by flow cytometry. Two‐sided Student's *t*‐test was used for intergroup comparisons. **p < 0.01. E2F3‐IT1‐SH: Jurkat cell transfected with short‐hairpin (SH) RNA with knocked‐down E2F3‐IT1. E2F3‐IT1‐NC: Jurkat cell transfected with empty vector, serving as negative controls

The lncRNA E2F3‐IT1 is located at chromosome 6, an intronic transcript of transcriptional factor *E2F3* (Figure S2A). Since the function of lncRNA is correlated with its subcellular localization, we carried out a cellular fractionation assay. The data indicated that the distribution of lncRNA E2F3‐IT1 is similar to the nuclear‐localized U6 snRNA and distinct from the cytoplasm‐enriched protein‐coding GAPDH mRNA (Figure S2B). We also carried out target prediction by using some bioinformatics tools, such as RNAInter.^6^ Of interest, lncRNA E2F3‐IT1 mainly interacts with transcription factors, histone modification, and RNA binding proteins (Figure S2C). Based on lncRNA E2F3‐IT1 subcellular localization and its predicted target moleculars, we inferred that the lncRNA E2F3‐IT1 acts its regulation effect mainly through transcription regulatory in the nucleus. Interestingly, the other two E2F3‐IT1 regulatory targets *PLSCR1* and *PARP9* were interferon response genes.^7^, ^8^ We proposed that E2F3‐IT1 acts its regulation effects mainly on the three interferon response genes (*LDLR, PLSCR1*, and *PARP9*) at the transcription level in the nucleus, while the exact functional mechanisms underlying the associations are still needed to be further elucidated.

In summary, the present study revealed a significant lncRNA–mRNA interaction network involved in RA and highlighted lncRNA E2F3‐IT1 as a novel functional lncRNA associated with RA pathogenesis. These results further elucidate the important roles of lncRNA in RA and provided insights into the diagnosis, classification, and treatment for RA. To our knowledge, this study represents the first effort to explore the lncRNA role on RA by integrating the evidence from multi‐omics data.

## CONFLICT OF INTEREST

The authors declare no conflict of interest.

## AUTHOR CONTRIBUTIONS

Long‐Fei Wu, Xing‐Bo Mo, Jia‐Hui He, Pei He, and Xin Lu recruited the patients and conducted the experiments. Long‐Fei Wu, Xing‐Bo Mo, and Jia‐Hui He wrote the manuscript and analyzed the data. Hong‐Wen Deng, Fei‐Yan Deng, and Shu‐Feng Lei revised the manuscript. Fei‐Yan Deng and Shu‐Feng Lei designed and supervised the study. All authors read and approved the final manuscript.

## ETHICS APPROVAL AND CONSENT TO PARTICIPATE

The study protocol was approved by the ethical committees of Soochow University. All study participants provided their written consent for participation in the study.

## DATA AVAILABILITY STATEMENT

The data that support the findings of this study are available from the corresponding author upon reasonable request.

## Supporting information

Supporting Information

Supporting Information

Supporting Information

Supporting Information

Supporting Information

Supporting Information
