# Supplementary material for: Integrative lncRNA–mRNA co‐expression network analysis identifies novel lncRNA E2F3‐IT1 for rheumatoid arthritis
Source: Clin Transl Med. 2021 Feb 24;11(2):e325. doi: 10.1002/ctm2.325 (PMC7905107; doi:10.1002/ctm2.325)
Supplement: Supplementary file 5 — Supporting Information [file CTM2-11-e325-s003.docx]

**Supplementary Table S5: The primers used in RT-qPCR**

| **Primer** | **Sequence (5'to3')** |
| --- | --- |
| IL-1β-forward | ATGGCAGAAGTACCTAAGCTCGC |
| IL-1β-reverse | ACACAAATTGCATGGTGAAGTCAGTT |
| IL-2-forward | CATTGCACTAAGTCTTGCACTTGTCA |
| IL-2-reverse | CGTTGATATTGCTGATTAAGTCCCTG |
| IL-4-forward | CGGCAACTTTGACCACGGACACAAGTGCGATA |
| IL-4-reverse | ACGTACTCTGGTTGGCTTCCTTCACAGGACAG |
| IL-6-forward | ATGAACTCCTTCTCCACAAGCGC |
| IL-6-reverse | GAAGAGCCCTCAGGCTGGACTG |
| IL-8-forward | ATGACTTCCAAGCTGGCCGTGGCT |
| IL-8-reverse | TCTCAGCCCTCTTCAAAAACTTCTC |
| IL-10-forward | AAGCTGAGAACCAAGACCCAGACATCAAGGCG |
| IL-10-reverse | AGCTATCCCAGAGCCCCAGATCCGATTTTGG |
| TNF-α-forward | GGCTCCAGGCGGTGCTTGTTC |
| TNF-α-reverse | AGACGGCGATGCGGCTGATG |
| IFN-γ-forward | GCATCGTTTTGGGTTCTCTTGGCTGTTACTGC |
| IFN-γ-reverse | CTCCTTTTTCGCTTCCCTGTTTTAGCTGCTGG |
| GAPDH-forward | GAAGGTGAAGGTCGGAGT |
| GAPDH-reverse | CTTCTACCACTACCCTAAAG |
