# Supplementary material for: Integrative lncRNA–mRNA co‐expression network analysis identifies novel lncRNA E2F3‐IT1 for rheumatoid arthritis
Source: Clin Transl Med. 2021 Feb 24;11(2):e325. doi: 10.1002/ctm2.325 (PMC7905107; doi:10.1002/ctm2.325)

**Supplementary information**

**Materials and Methods**

**Study subjects and sample preparation**

A total of 43 subjects (25 RA patients and 18 healthy controls) were recruited for lncRNA and mRNA microarray expression profiling in PBMCs. Another independent sample set, including 70 subjects (35 RA patients and 35 healthy controls), was recruited for validation of selected lncRNA and mRNA expression (**Table 1a**). All RA patients satisfied the 1,987 diagnosis criteria of the American College of Rheumatology, and had an examination of tender and swollen joints and disease activity score of 28 joints (DAS28) recorded. Laboratory investigations included C-reactive protein (CRP) and erythrocyte sedimentation rate (ESR). Active RA was defined as having a DAS28 of 2.6 or higher. The healthy controls were excluded for any of the following diseases: severe cardiovascular diseases, liver and kidney dysfunction, malignant tumor and other immune diseases including systemic lupus erythematosus (SLE), ankylosing spondylitis. No significant differences in age and BMI were detected between the RA cases and controls in the above two study samples. The study was approved by the ethical committee of Soochow University (No: 2012-146). All the study subjects signed informed consents before enrollment.

Peripheral blood samples were obtained from RA patients and healthy controls. The PBMCs were isolated from 15 ml peripheral blood by density gradient centrifugation using lymphoprep (Greiner bio-one) within 4 hours after phlebotomy. Total RNA was isolated from PBMCs by standard phenol-chloroform extraction using Trizol reagent (Invitrogen) according to the manufacturer’s instructions, and the concentration was measured by Nanodrop Spectrophotometers (Thermo Fisher Scientific). RNA quality was checked on Bioanalyzer Nanochip (Agilent Technologies). Genomic DNA was isolated from 18 RA patients and 25 healthy controls using genomic DNA extraction kits (Qiagen), and DNA integrity was analyzed by agarose gel electrophoresis.

**Transcriptome-wide lncRNA and mRNA expression profiling**

About 2.0 μg of total RNA extracted from PBMCs was used to profile transcriptome-wide mRNA expression by using lncRNA&mRNA Human Gene Expression Microarray V4.0 (CaptialBio, Beijing, China) for each subject. Total RNA was reverse-transcribed, and then the double-stranded cDNAs were synthesized using the CbcScript reverse transcriptase with cDNA synthesis system, according to the manufacturer's protocol (CaptialBio). Microarrays were scanned with a confocal LuxScan™ scanner. Scanning settings were adjusted across arrays. Raw data was extracted by Agilent Feature Extraction (V_10.7_) and was normalized using GeneSpring GX program (V_12.0_). The probes with detection rate less than 80% and/or incomplete annotation information were filtered. Then, log_2_ transformation was applied to the data by using the adjust data function of Multi-experiment Viewer (MeV) software. Expression data between RA patients and healthy controls was compared using *t*-test.

**Construction of co-expression network by using WGCNA**

Based on PBMCs microarray dataset, co-expression network of lncRNA and mRNA was constructed by the R package “WGCNA”^1^. Gene network was constructed to identify co-expression genes via WGCNA. In addition, co-expressed mRNAs in the identified module were functionally annotated by using gene ontology (GO) and Kyoto Encyclopedia of Genes and Genomes (KEEG) pathway analysis. The enriched genes in specific GO terms and lncRNAs that were strongly correlated (correlation coefficient r^2^ ≥ 0.8) with these protein coding genes in each module were sorted out. Then sub-co-expression modules of this lncRNAs and mRNAs were constructed and hub RNAs in the sub modules were determined, based on the number of connectivity of the RNAs with other RNAs in each module.

**Real-time quantitative PCR (RT-qPCR)**

For the PBMCs sample preparation, 15 ml peripheral blood samples were collected and stored in vacuum blood collection tubes containing sodium citrate. PBMCs were separated by density gradient centrifugation using lymphoprep (Greiner bio-one). Then, total RNA was purified from PBMCs by using Trizol reagent (Invitrogen). For cultured cells sample, cells were washed with PBS for three times before 1ml Trizol was added. Total RNA from PBMCs or cell tissues was reverse transcribed into cDNA. The cDNA served as template for amplification by qPCR using SYBR Green. The cDNA amplifications were monitored using ABI 7900 under 1 cycle of pre-degeneration at 95°C for 10 min, 40 cycles of amplification at 95 °C for 10s and 60 °C for 20s. This assay was carried out in triplicate for each sample, plus a blank control without template. The relative quantity (RQ) of the gene expression in each sample was calculated by normalizing to house-keeping gene GAPDH. Data were analyzed using the 2^-△△CT^ method. The sequences of primers in RT-PCR were showed in **Supplementary Table S5**.

**Sub-cellular fractionation assay**

To study sub-cellular distribution of lncRNA, cytoplasmic and nuclear fractions of Jurkat cells were isolated and examined lncRNA expression separately using RT-qPCR. Briefly, a total of 1×10^7^ Jurkat cells in T-flask were washed with PBS and then incubated in hypotonic buffer on ice. After 5 min of centrifugation at 2,000 g, the supernatant was collected as the cytoplasmic fraction. The remainder was washed with PBS and resuspended in lysis buffer to obtain the nuclear fractions.

**Lentiviral construction, production and transfection**

Lentiviral vectors harboring E2F3-IT1 sequence or empty vector were constructed and stably transfected into Jurkat T cells. Jurkat cells were grown in RPMI 1640 culture medium (Hyclone) supplemented with 10% fetal bovine serum (FBS), 1% penicillin-streptomycin (TransGen). HEK293T cells were cultured in DMEM (Hyclone) supplemented with 10% FBS and 1% penicillin-streptomycin. Gene knock-down sequence was synthesized and then cloned into pLVTHM lentiviral vector. Sequenced successfully constructed plasmids were transfected into the prepared competent cells. Virus were harvested from the medium of HEK293T cells and transfected into Jurkat cells. The transfection efficiency was assessed by RT-qPCR according to standard protocols. Specifically, the target sequence of lncRNA E2F3-IT1 was TCCTCATCTCTTGCAGTGA. The expression of lncRNA E2F3-IT1 was validated by RT-qPCR with the following primers: 5’-CGATGGAGAAAGCAATGGCG-3’ (forward) and 5’-ACCAACTTTTCCACAACATG-3’ (reverse).

**Cell proliferation assay**

Cell proliferation was measured with CCK8 assay. For cell proliferation test, 5.0×10^3^ cells/well were seeded in 96-well plate. lncRNA E2F3-IT1 knock-down (E2F3-IT1-SH) and negative control (E2F3-IT1-NC) cells at the logarithmic growth phase were cultured for 0h, 24h, 48h and 72h. After incubation, 10 μl of CCK8 solution was added into each well and incubated at 37°C for 1 h. Absorbance at the wavelength of 450 nm was examined to indicate the number of surviving cells.

**Cell cycle assay**

For cell cycle assay, E2F3-IT1-SH and E2F3-IT1- NC cells were labeled with propidium iodide (PI) using a Cell Cycle Assays Kit (Beyotime, China) according to the manufacturer’s protocol. Briefly, cells were harvested, washed twice with cold PBS and fixed with 70% ice-cold ethanol for 2 hrs at a density of 1×10^5^ cells/ml. The fixed cells were subsequently treated with 200 μg/ml ribonuclease for 30 min at 37°C. Then PI was added to the cell cultures at a final concentration of 50 μg/ml. DNA content analysis shows the percentage of cells within different phases of the cell cycle, i.e, increased amount of 4N-DNA content cells representing G2/M cell cycle arrest. The DNA content was quantified by flow cytometry with an excitation wavelength of 488 nm and an emission wavelength of 625 nm. The data were analyzed using BD Accuri C6 software.

**Cell apoptosis assay**

The cell apoptosis was assessed using Annexin V Apoptosis Detection Kit (BD Biosciences). The stably transfected cells were harvested by centrifugation, washed twice by PBS and stained with Annexin V and 7AAD according to the manufacturer’s instructions. After incubation in the dark for 15 min at room temperature, cells were resuspended with 1× Annexin V binding buffer and examined by flow cytometry. Early apoptotic cells (Annexin V^+^/7AAD^-^), late apoptotic cells (Annexin V^+^/7AAD^+^), and total apoptotic cells with fluorescence Annexin V^+^/7AAD^-^ and Annexin V^+^/7AAD^+^ were calculated.

**Cell activation assay**

For cell activation, E2F3-IT1-SH and E2F3-IT1-NC cells at the logarithmic growth phase were seeded in 6-well plate and treated with or without 5 μg/ml Phorbol-12-Myristate-13-Acetate (PMA). After incubation for 12 h at 37°C, cells were harvested and washed twice with cold PBS before fixing in 1% paraformaldehyde. After washed with PBS twice, cells were incubated with allophycocyanin (APC)-conjugated mouse anti-CD69 mAb for 30 min at 4°C protected from light. Finally, cells were washed and suspended in a staining buffer (PBS containing 1% BSA). CD69 expression was detected using flow cytometry and analyzed using FlowJo (Ashland) software.

**Statistics**

Continuous data were presented as mean and standard deviation. Two-sided Student’s *t*-test was used to test the difference in mean between two groups. All statistics were performed by SPSS 19.0. All the statistical plots were generated using GraphPad Prism 4.0 or R software.

**REFERENCES**

1. Langfelder P, Horvath S. WGCNA: an R package for weighted correlation network analysis. *BMC Bioinformatics.* 2008;9:559.

**Supplementary Figure legends**

**Supplementary Figure S1** Flow chart for this study.

**Supplementary Figure S2** (A) Diagram of lncRNA E2F3-IT1 location. (B) Sub-cellular distribution of E2F3-IT1 in Jurkat cells detected by RT-qPCR. The RT-qPCR data, represented as a percentage of the detected transcripts in whole cell fraction. U6, a canonically nuclear-associated non-coding RNA, and GAPDH, a protein-coding mRNA, were assessed as controls. Data are presented as means ± SD. (C) The top 100 interactions of lncRNA E2F3-IT1 predicted by RNAInter.


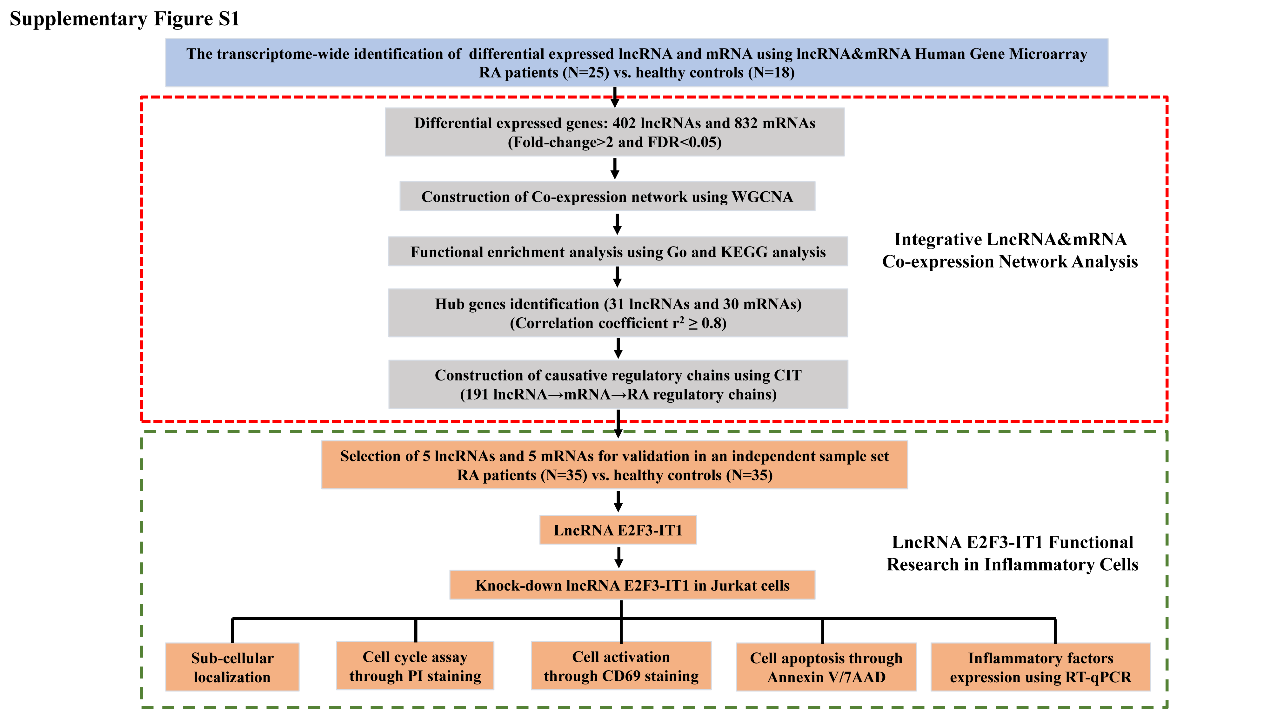


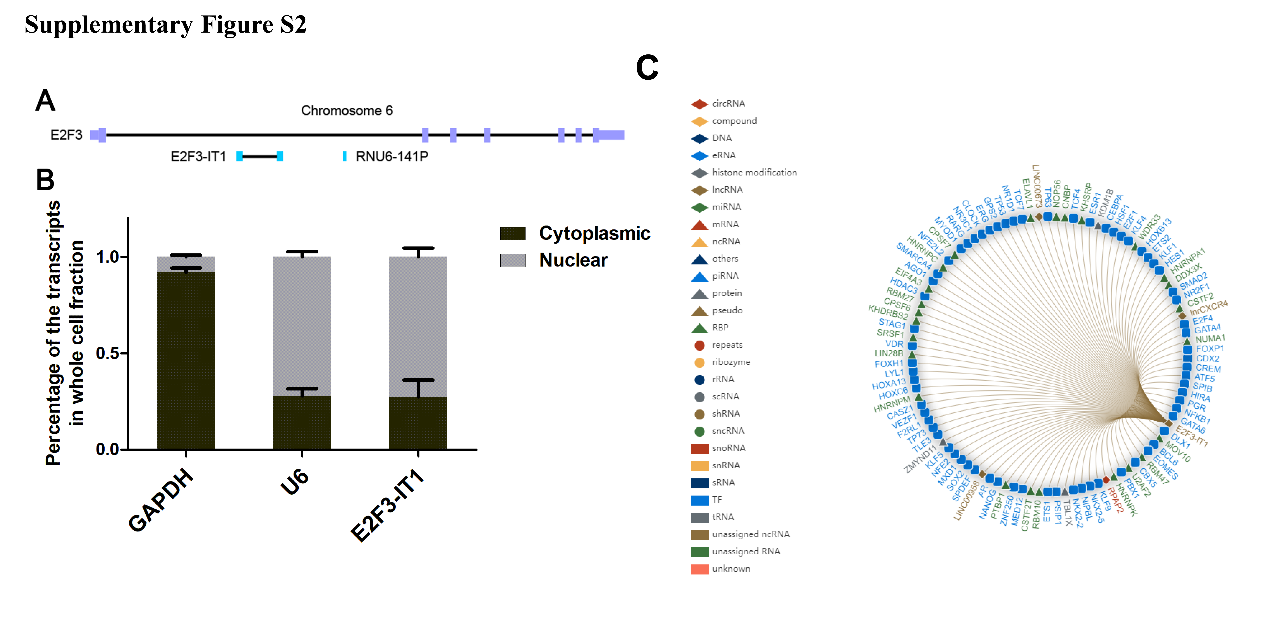

Supplement: Supplementary file 6 — Supporting Information [file CTM2-11-e325-s005.docx]
